# Comparing medicine and management: methodological issues

**DOI:** 10.1186/s12913-016-1390-x

**Published:** 2016-05-24

**Authors:** V. Burau

**Affiliations:** Department of Political Science, Aarhus University, Aarhus, Denmark

**Keywords:** Medicine and management, Cross-country comparison, Methods, Multi-level comparisons, Institutional explanations

## Abstract

**Background:**

In the study of medicine and management, there is a strong interest in cross-country comparison. Across healthcare systems in industrialised countries, New Public Management has provided a similar reform template, but new governing arrangements exhibit significant national variations. The comparative perspective also offers a leverage to overcome the resistance focus of earlier studies. Comparison raises two overall questions: in what similar and different ways are relations between medicine and management changing across industrialised countries? Why is change occurring in different ways? The questions reflect exploration and explanation as the two basic rationales for comparison.

**Methods:**

The aim was to provide a critical discussion of different approaches to comparing medicine and management across countries. The analysis was based on a narrative review of relevant studies from several bodies of literature.

**Results and discussion:**

The majority of studies exploring medicine and management adopt macro level approaches to comparison. Studies draw on a range of notions, including area specific ideal types of professionalism, professionalism as countervailing powers and governmentality. There are much fewer studies exploring relations between medicine and management at the meso level. Analyses treat comparison as a two-dimensional exercise looking across both countries and levels. The majority of studies draws on institutional explanations. These are variations of the path dependency argument and studies include both sector specific and broader political and administrative institutions. There is an emerging body of process-based explanations which connect macro level institutions to organisations and which promote more non-linear comparisons.

**Conclusion:**

The lack of meso level comparisons drawing on process explanations is problematic. Empirically, we need to know more about how relations between medicine and management are different across countries. Theoretically, we need to better understand how we can transpose analytical insights from institutional explanations at macro level to studies that are multi-level and also include the meso level of organisations. Methodologically, we need to address the challenges arising from more non-linear approaches to comparison, especially how to organise close international research collaboration over an extended period of time.

## Background

In the study of medicine and management, there is a strong interest in cross-country comparison, especially for two reasons. Across healthcare systems in industrialised countries, New Public Management has provided a similar reform template, but new governing arrangements exhibit significant national variations [1–6]. For example, in their study of changes in professional self-regulation in the Germany and the United Kingdom, Kuhlmann and colleagues [7] find that the common trend towards network governance plays out differently between the two countries; governance arrangements are more exclusionary in Germany and more pluralist in the United Kingdom. Dent and colleagues [8] make a similar observation in their case study of hospital modernisation in a North German state. The emergence of a distinct ‘New Steering Model’ is compatible with the strong German tradition of *Rechtsstaat*, but represents a minimalist approach to modernisation compared to the United Kingdom. National variations offer rich material for comparison and also raise questions about how to explain the specific interplay between international trends and country specific contexts that drives the process of translation. Studies of medicine and management in the literature on comparative health policy for instance argue that differences in institutions like the healthcare and political system shape the specific choice of governing instruments. This helps Giamo [4] to explain why market reforms in Britain, Germany and the United States varied in goals, targets, the scope of competition and impacted differently on the medical profession. Similarly, Burau and Vrangbæk [9] can account for the specific balance between hierarchy and professional self-regulation in the governance of medical performance in four European countries.

The cross-country comparative perspective also offers a leverage to overcome the resistance focus of earlier studies of medicine and management [10, 11]. The view across countries highlights the embeddedness and diversity of relations between medicine and management. Kirkpatrick and colleagues [5] powerfully underscore this in their comparison of the impact of hospital reforms in Denmark and England. Although both countries have National Health Services and introduced reforms around the same time, the consequences for the relative re-stratification of medicine varied considerably. Comparison helps moving beyond ideal typical hypotheses at macro level and towards more contextualized, qualitative and process oriented approaches to analysing the control of doctors [11]. Studies use the context sensitivity of comparison as a leverage, for example, to expose critical differences in changes in professional governance [7] and to highlight the relative capacity of the medical profession to transform itself and to incorporate changes inspired by New Public Management [10]. In short, within the study of medicine and management, comparison can contribute with a more critical analysis and identify country specific differences within overall similarities. This echoes the salient contribution of comparison, to avoid false generalisations (‘medicine and management in all countries are the same’) and false uniqueness (‘medicine and management in this country are like no other’).

For the study of medicine and management, comparison raises two overall questions: in what similar and different ways are relations between medicine and management changing across industrialised countries? Why is change occurring in different ways across countries? The questions reflect the two basic rationales for comparison: exploring and explaining similarities and differences [12–14].

The aim of the present article is to provide a critical discussion of different approaches to comparing medicine and management across countries. Studies in the field originate from several bodies of literature: comparative health policy, comparative public administration, and comparative studies of the professions, both from the sociology of professions and the organisational studies traditions. Considering the highly diverse nature of the literature and the aim of mapping and analysing central conceptual tools of comparison in the field, the search and synthesis strategies associated with a narrative review appeared to be most suitable. The focus was on sketching out broad narratives about how to compare medicine and management across countries to capture the complexities and ambiguities inherent in the debate. In terms of search strategies, following the author’s expertise in the field, studies were initially identified based on personal knowledge and snowballing. This was complemented with a search in relevant databases. The focus was on studies which had a primary interest in relations between management and medicine and which (more or less explicitly) adopted a comparative perspective. In terms of synthesis strategies, the main goal was to identify different conceptual approaches for comparing medicine and management across different countries. Several rounds of thematic mapping helped to identify the central themes and concepts across studies and led to the following organising framework. It has two dimensions, exploration and explanation, with two sets of variations. In terms of exploration studies varied depending on if they focused on the macro level of the healthcare, administrative or political system or on the meso level of healthcare organisation such as hospitals and primary care providers. In terms of explanation, studies varied depending on if they drew on institutional explanations or on explanations related to processes.

## Results

### Exploring relations between medicine and management

Macro level comparisons are a well-established approach to comparing relations between medicine and management in the literature on comparative health policy [15]. Comparative studies analyse a variety of issues, from how the state and medical interest organisations negotiate healthcare reforms to how healthcare reforms affect the medical profession in terms of their professional organisation and development and their relative autonomy. Examples of the first type of study include Giamo’s [4] analysis of the influence of the medical profession on market reforms in Britain, Germany and the United States. Another example is Essen’s [16] comparison of the strategies used by the medical profession in the Netherlands, Germany and England in connection with the introduction of new hospital payment systems. The Dutch medical profession was willing to solve conflicts, while their counterparts in England and Germany chose more confrontational strategies. The author argues that these significant differences reflect specific institutional and interest configuration typical of the individual countries. Other studies focus on arrangements for governing the medical profession. Moran and Wood [17] examine how the healthcare systems in Britain, Germany and the United States have shaped the regulation of doctors in terms of development, organisation, process and outcomes. Kirkpatrick and colleagues [5] make a similar point in their comparative analysis of how recent hospital reforms have impacted on the professional development of medicine and its re-stratification. The emergence of an administrative élite has been stronger in Denmark than in England, reflecting differences in the opportunities for private practice and the closeness of state-profession relations.

Within the sociology of professions, comparative studies of profession-state relations are another example of macro level comparisons [18]. The focus is on analysing the professional project of medicine and in what ways states through their healthcare systems and health policies influence this project. The historical perspective has been a major driving force and studies identify different types of area specific professionalism. Collins [19] distinguishes between two types: an Anglo-American ideal type of professionalism, which is characterised by self-employed practitioners who operate in a market of services and who are free to control their own work conditions; and a Continental ideal type, whereby professions are part of state development and become elite administrators by virtue of credentials. Light [20] moves away from ideal types and instead emphasizes the time specific nature of medical professionalism in his concept of ‘countervailing powers’. His comparative analysis across a range of industrialised countries highlights the specific dynamics that occur in a two dimensional space of doctor-state relations and the relative dependence on doctors’ employment. For example, hospital consultants in the United Kingdom, came under closer influence of the state between 1980 and 1992, reflecting the introduction of the internal market in the National Health Service. Some studies go further; drawing and the Foucauldian notion of governmentality, they emphasise the independent relations between professions and states and how professional projects of medicine are inextricably linked to larger or smaller projects of governing the healthcare state. Johnson [21] argues that professions like doctors are an integral part of governing as they respond to specific liberal governing problems. The science base of liberal government requires autonomous expertise to legitimise state actions and expertise becomes institutionalised. Pickard [22] shows, for instance, that the development of geriatric medicine in the United Kingdom after the Second World War was predicated not only on the availability of new knowledge about the possibilities to treat older people, but also on the use of this knowledge for discharge older people from long-stay beds.

Over the last decades, the emerging focus on governance has challenged the exclusive concern for the macro level of relations between medicine and management. In the practice of governance, processes of political-administrative decision making are more decentralised and closely intertwined with meso level processes of introducing and maintaining change in public services [23, 24]. Governance sets focus on both formal and operational policy [25]. There are a number of studies that use the macro level of healthcare systems as a stepping stone for meso level analyses of governing medicine and management across different countries. For example, in their comparative study of changes in professional governance Kuhlmann and colleagues [7] choose Germany and the United Kingdom as two different healthcare systems that are likely to make for distinct institutional pathways. Within this, the authors focus on standards, benchmarks and clinical guidelines as meso level governance practices, and examine both top-down policies and bottom-up developments from within the medical profession. This turns comparison into a two dimensional endeavour, which explores medicine and management across countries and across different yet nested levels. Following Clarke [26], this can be thought of as a ‘conjunctural analysis’ which explores the unsettled formations that connect different levels of governance and which highlights the potentially contradictory forces, pressures and tendencies which are at play at the same moment in time.

There are also some examples of this type of multi-level comparison in the literature on organisational studies of the professions. Kuhlmann and colleagues [10] for instance compare new modes of control in hospitals in seven European countries by looking at contexts of hospital governance at both national and organisational levels. They argue that governance arrangements increasingly combine elements of management and professional self-regulation and they identify three patterns of control: integrated control (Denmark, Netherlands), partly integrated control (Spain and Germany) and fragmented control (Portugal, Poland and Greece). In their study of hospital modernisation in a North German state, Dent and colleagues [8] also treat the specific hospital as a local variant of an archetype, which is revealed by its relations to organisational and (sub)national institutional contexts. Hartley and Kautsch [27] in their comparative study of doctors’ engagement with hospital management choose Poland and the United Kingdom because of their different healthcare systems and use these to frame their organisational analysis. This offers an implicit test of how strongly changes at the level of hospitals are filtered through macro level institutional contexts.

In summary, macro-level studies dominate the comparison of medicine and management. Studies originate from the literature on comparative health policy and from the sociology of professions. There is an emergent body of comparisons of medicine and management, which adopts a multi-level perspective.

### Explaining relations between medicine and management

The distinction between macro and meso level explorations of relations between medicine and management in different countries in part corresponds to a distinction between institutional and process-based explanations of the differences and similarities found.

Macro level comparisons of relations between medicine and management typically focus on institutional explanations. This builds on variants of the path dependency argument [28]. Institutions shape the interests of key stakeholders in medicine and management as well as their resources and through this also allocate power. Relations between medicine and management emerge as institutionally grounded [15]. This is illustrated by Giamo’s [4] comparative study of medicine and the politics of market reforms in Britain, Germany and the United States. She describes this as an interplay between actors and institutions in both broader political and sector specific arenas. Institutional contexts determine the presence or absence of certain actors and affect governments’ capacity to control especially the medical profession; this offers a leverage to realise more or less radical market reforms. The centralised decision making in Britain offered the government the possibility to unilaterally decide the terms of the reform legislation, while the decentralised decision making in Germany gave sectoral interests including the medical profession ample possibilities to influence the legislative process. Such interests need to have the ability to take collective action and this is what the medical profession in the United States lacked in the legislative process leading to the Clinton plan. It was organisationally fragmented and it did not have a clear policy stance.

Studies in the literature on comparative health policy focus on the healthcare system as a set of sector specific institutions. Kuhlmann and colleagues [7] use healthcare systems as ideal types in their comparative study of changes in professional governance; healthcare systems ‘provide alternative blueprints for the development of social healthcare for citizens in western countries’. Different constellations of stakeholders in the National Health Service in the United Kingdom and the Social Health Insurance Model in Germany shape new forms of governing professionals in distinct ways. Burau and Vrangbæk [9] rely on a related typology of healthcare states initially developed by Michael Moran to capture a set of regulative institutions related to consumption and provision as central areas of governing healthcare. The first set of institutions includes mechanisms for deciding on the total volume of financial resources for healthcare and for giving patients access. The second set of institutions concerns mechanisms for governing provider organisations. Essen [16] treats the institutional context of the healthcare system more loosely in her comparative study of the introduction of new hospital payment systems. The healthcare system consists of a set of institutions, including: economic autonomy, corporate bodies, decision making and state-profession relations. Other studies include broader political institutions. In her comparative study of markets and medicine in Britain, Germany and the United States Giamo [4] distinguishes between two sets of institutions: those related to state structures like the formal political institutions and electoral systems which give governments more or less autonomy from societal interests; and those related to interest groups and the organisational characteristics of medical associations. This is similar in studies inspired by the literature on comparative of public administration. Their focus is on broader political administrative institutions like structures and traditions of public administration. For example, following Hood, Dent [2] examines in what ways public management traditions can explain differences in changes to hospital management. The author includes the United Kingdom with its pragmatic traditions, Germany with its law oriented tradition typical of Continental Europe, and Italy with its scientific tradition also found in the United States. The pragmatic and scientific traditions have offered a leverage for introducing a quasi-market organisation in the National Health Services of Italy and the United Kingdom, while legalistic traditions have only allowed for a piecemeal adoption of such reforms in Germany.

Comparative studies of professions also identify a range of general and sector specific institutions. Writing from the organisational studies tradition, Kirkpatrick and colleagues [6] point to a variety of dimensions of national institutional contexts in their study of how a particular US model of hospital management has been translated into four European countries. This includes: the relative strength of professional dominance and the extent to which the medical profession controls health related institutions, services and funding; the nature of administrative cultures and the particular status of medical professionals as salaried employees or civil servants; and the wider political governance of public services and the specific distribution political-administrative control over hospitals. The earlier historically oriented studies identify more deeply embedded institutions, such as geographically and historically specific state formations. Heidenheimer [29] shows how a strongly bureaucratic state in Germany incorporated doctors in large numbers and gave professional organisations quasi-official status. Johnson [21] focuses on the modern liberal state, which institutionalised expertise to address its governing problems.

Institutionally based explanations make a more or less implicit assumption that organisations are affected by macro level institutional contexts by virtue of being part of them [6]. Dent and Bode [3] refer to the connections between levels as ‘procedural contingency’ and it varies from country to country and includes both national path dependencies and sub sectoral dynamics. Kuhlmann and colleagues develop a more specific approach to systematically apply the cross country comparative perspective to the level of the organisation. Their ‘Hospital Control Assessment Framework’ consists of five components: (1) Characteristics of the healthcare state and institutional contexts of hospital governance, (2) governance structures of the hospital, (3) financial controls, (4) quality and safety controls, and (5) medical self-governing controls. The first two components are structural, whereas the remaining components combine actor-centred with organisational dimensions, asking who is responsible at what level of the organisation.

Process-based explanations take the actor perspective further and look more closely at the specific ways in which organisations connect to institutions at the macro level by drawing on institutional theory [1, 2]. It sees organisations as nested in organisational fields, including the healthcare, administrative and political systems. Process-based explanations help to fill in details about the dynamics at play and to account for the variable ways in which medicine and management in healthcare organisations interpret and adapt (international) reform templates. This was the focus of the COST Action IS0903 on ‘Enhancing the role of medicine in the management of European health systems: implications for control, innovation and user voice’. The perspective was comparative, but many studies emerging from the network remained single-country (see for example the special issue edited by Dent and Bode [3]). One notable exception is the study by Kirkpatrick and colleagues [6]. They focus on the notion of translation in their comparative study of how a particular model of hospital organisation first introduced in the United States came to be implemented in four European countries. The basic idea is that national and organisational institutional contexts offer editing rules, which influence how reform templates are legitimated and enacted in specific national and local settings. These rules allocate varying potential for agency on different levels and result in differences in perceptions about the desirability and feasibility of the particular reform model of hospital management. For example, national healthcare systems vary in terms of how desirable and feasible radical reform is considered and editing rules may be more prescriptive (like in the English NHS) or less prescriptive (like in the French and Italian healthcare systems) as a result [6]. This process of fitting generic concepts to national and local contexts helps to explain how similar reform templates become heterogeneous when implemented.

Process-based explanations also alter the nature of cross country comparison; it becomes much less linear, whereby similarities and differences cannot simply be read off from macro institutional contexts and their respective ideal types. Instead similarities and differences emerge from a process which involves both multiple levels and local actors. For example, based on their analysis of hospital modernisation in a North German state, Dent and colleagues [8] conclude that institutional fields are typically in movement and that change is negotiated and mediated by all stakeholders. Kirkpatrick and colleagues [6] further illustrate the variability of this process in their comparative analysis of translating models of hospital management. Editing rules exist at both national and local institutional contexts and they are implicit rather than prescriptive. Comparison emerges as an endeavour which involves bringing context in. This is echoed by more constructivist approaches to comparison. One example is the so-called ‘decentred’ approach to comparison [30], which promotes context sensitivity in two ways. Conceptually, it focuses on the social organisation of healthcare and uses professions as the meso level stepping stone for multi-level comparisons. This moves comparison beyond the macro level of the healthcare, administrative and political system and related assumptions that processes of change across different countries occur in a linear way. Methodologically, the decentred approach to comparison sees the research team as having distributed expertise that is socially constructed. The implied synergy requires research teams that are international and interdisciplinary. In the research team, the collaborative effort extends to all stages of the research process. It includes formulating the initial research interest, defining key conceptual terms, analysing the research results and comparing across country cases. Being sensitive to context means that expertise emerges in the process of interaction and it also offers the primary leverage for generalisations. For example, Kuhlmann and colleagues [10] applied this approach to comparison in their study of new controls of hospitals. Their research team included scholars from seven countries, with wide ranging expertise from health management, economics and medicine to sociology and political science, and from backgrounds in policy, practice and research. The team developed a conceptual framework and jointly analysed the comparative material as part of series of workshops and virtual meetings. This offered an opportunity for in depth discussion and validation of both indicators and findings.

In summary, instutional explanations based on variations of the path dependency argument dominante comparisons of medicine and management. Studies originate from a wide range of literature and identify a great variety of different institutions. Only very few studies draw on process based explanations and look more closely at how organisations connect to institutions. This type of explanation also changes the nature of comparison, which becomes less linear. Country cases can no longer be positioned on a given continuum, based on pre-determined dimensions for comparison. Instead, comparisons are less predictable and emerge as part of close, international research collaborations which stretch over an extended period of time.

## Discussion

The majority of studies exploring relations between medicine and management across different countries adopts macro level approaches to comparison and draws on institutional explanations. This commonality in overall perspective hides considerable diversity in terms of research questions addressed and definitions of institutions. For example, in the literature on comparative health policy, studies address questions about how state and medical interest organisations negotiate healthcare reforms, while in the literature on the sociology of professions the main concern is how the state through its healthcare system and policy influences the professional project of medicine. In terms of explanation, studies refer to both sector specific and broader political and administrative institutions. A prominent example of the former is the healthcare system, whereas examples of the latter include state structures, administrative cultures and interest group arrangements. The diversity in research questions and institutional explanations reflects the variety of disciplines studies draw on, ranging from political science and public administration to social policy and sociology. This makes it potentially difficult to systematically relate research findings to each other.

There are also much fewer studies exploring relations between medicine and management across countries at the meso level of organisations: ‘[m]ost empirical research [at the organisational level] has been based on single case studies, with only few analyses based on multiple, comparative or longitudinal case studies’ [11] (for an overview see [31]). Such analyses originate from the literature on welfare governance and organisational studies of the professions and they treat comparison as a two-dimensional exercise looking across both countries and levels. Studies draw on process-based explanations, such as the Hospital Control Assessment Framework and approaches focusing on processes of translation.

In terms of future research, there is a need for more meso level, comparative studies of medicine and management. Public sector reforms over the last decades have significantly decentralised governing arrangements and the organisation level has become key for understanding relations between medicine and management per ser. Meso level studies also address questions that are central for understanding how relations between medicine and management can contribute to the future developments of healthcare systems. This includes questions about the strategies doctors use [32, 33] to contribute to organisational change and what mechanisms lead to the acceptance (or resistance) of public management reforms [11].

Theoretically, this requires exploring ways how to transpose analytical insights from institutional explanations at macro level to studies that are multi-level and also include the meso level of organisations. This involves broadening our understanding of contexts as something that is also local and organisationally specific, while accepting a nested view of context that allows reconnecting to national level institutions [34].

Methodologically, meso level, comparative studies of medicine and management promote more non-linear comparisons that are sensitive to a range of contexts across countries and levels. The decentred method of comparison offers a more systematic account of the specific steps involved, but raises its own challenges. For example, a true (theoretical and empirical) synthesis of detailed knowledge of countries and organisations, requires an international research team, that has the possibility to collaborate over an extended period of time.

## Conclusion

In the study of medicine and management, there is a strong interest in cross-country comparison. Across healthcare systems in industrialised countries, New Public Management has provided a similar reform template, but new governing arrangements exhibit significant national variations. The comparative perspective also offers a leverage to overcome the resistance focus of earlier studies. Comparison raises two overall questions: in what similar and different ways are relations between medicine and management changing across industrialised countries? Why is change occurring in different ways? The questions reflect exploration and explanation as the two basic rationales for comparison. The aim was to provide a critical discussion of different approaches to comparing medicine and management across countries. The analysis was based on a review of relevant studies from several bodies of literature: comparative health policy, comparative public administration, and comparative studies of the professions, both from the sociology of professions and the organisational studies traditions.

The majority of studies exploring medicine and management adopt macro level approaches to comparison. Studies draw on a range of notions, including area specific ideal types of professionalism, professionalism as countervailing powers and governmentality. There are much fewer studies exploring relations between medicine and management at the meso level. Analyses treat comparison as a two-dimensional exercise looking across both countries and levels. The majority of studies draws on institutional explanations. These are variations of the path dependency argument and studies include both sector specific and broader political and administrative institutions. There is an emerging body of process-based explanations which connect macro level institutions to organisations and which promote more non-linear comparisons. The lack of meso level comparisons drawing on process explanations is problematic. Empirically, we need to know more about how relations between medicine and management are different across countries. Theoretically, we need to better understand how we can transpose analytical insights from institutional explanations at macro level to studies that are multi-level and also include the meso level of organisations. Methodologically, we need to address the challenges arising from more non-linear approaches to comparison, which are more emergent and which are based on close, international research collaboration over a longer period of time.

### Ethics and consent

This article reviews published work and it was therefore not necessary to apply for ethics approval.
